# Secular trends in motor abilities of Xinjiang children and adolescents aged 7–18 years from 1985 to 2019

**DOI:** 10.3389/fpubh.2024.1419728

**Published:** 2024-12-12

**Authors:** Shuai Zhang, Chengyue Li, Almujiang-Yimitri Tarken, Weiming Li

**Affiliations:** ^1^Institute of Physical Education, Xinjiang Normal University, Urumqi, China; ^2^School of Physical Education and Health, East China Normal University, Putuo, China

**Keywords:** Xinjiang, physical fitness, secular trends, children, adolescents

## Abstract

**Objective:**

To assessment the secular trend in physical fitness of children and adolescents aged 7–18 years in Xinjiang from 1985 to 2019.

**Method:**

The data are derived from test scores of Xinjiang Chinese children and adolescents aged 7–18 years by the China National Student Health Monitoring Centre National Student Physical Fitness Monitoring in 1985, 1991, 1995, 2000, 2005, 2010, 2014, and 2019. The physical fitness indicators included speed, cardiorespiratory fitness, muscular strength, power, and flexibility.

**Results:**

The overall physical fitness of Xinjiang children and adolescents aged 7–18 in Xinjiang demonstrated downward trend from 1985 to 2019. During the period from 1985 to 1995, the speed, power, and cardiorespiratory fitness improved significantly but worsened from 1995 to 2005. The pace of decline in physical fitness slowed down from 2005 to 2014. Some components of physical fitness improved, but most components of physical fitness continued to worsen from 2014 to 2019.

**Conclusion:**

In conclusion, the overall physical fitness of Xinjiang children and adolescents aged 7–18 years worsened from 1985 to 2019, and some relatively positive trends have been found in recent years. Trends in physical fitness vary among children and adolescents by gender and age, and these differences should be emphasized in the development of relevant physical fitness policies as well as interventions.

## 1 Introduction

Physical fitness is the main indicator to measure the body's physical activity and physical exercise ability, and it can also help us clearly understand our health level (1–4). Many studies have shown that physical fitness is closely related to childhood obesity, cardiovascular and cerebrovascular diseases, nutrient intake levels, and body shape development. In addition, there is a strong relationship between physical fitness and height and weight gain in children and adolescents. Although the development of height in children and adolescents is genetically determined, there is also a relationship between physical fitness and height development (5–8). The child and adolescent growth and development and the development of physical fitness are also related to the regional economic level (9–11). Economic development can improve the living standards of children and adolescents, which is conducive to the development of physical shape and the improvement of physical fitness (9–11), but it also has some negative impacts. Some studies have shown that higher economic status and family income levels are positively correlated with obesity. An increase in income will reduce health levels by promoting the intake of obese foods and reducing the intake of vegetables, fruits, and organic grains (12–14). The development of the economy has also changed the way residents travel, increasing sedentary time, which is not conducive to the development of the physical fitness of children and adolescents (15, 16). Previous studies also showed that the physical fitness of children and adolescents aged 7–18 years in Xinjiang continued to decline from 1985 to 2014 (17). The flexibility of Chinese rural children and adolescents has improved, power and muscular strength continued to decline from 2010 to 2019. Speed, flexibility, and muscle strength rebounded, and power continued to decline for rural girls (18, 19). However, there is little relevant research on trends in physical fitness among children and adolescents in Xinjiang in recent years. In addition, previous studies have focused on the secular trend in physical fitness for children and adolescents in Xinjiang, while sex and age differences are less discussed.

We assumed that the physical fitness of Xinjiang children and adolescents showed positive trends in recent years and that there were sex and age differences. Therefore, by the Chinese National Surveillance on Students' Constitution and Health (CNSSCH) from 1985 to 2019, we aim to investigate (1) secular trends in physical fitness for Xinjiang children and adolescents, (2) the changes between subgroups (sex and age) to find inequalities in the health of Chinese children and adolescents and (3) how trends in physical fitness compare to China as a whole and to other developed and developing countries.

## 2 Materials and methods

### 2.1 Study subjects

The data used in this paper comes from the successive National Student Physical Fitness and Health Research Reports (20–26). The CNSSCH was organized by the Ministries of Education, Health, Science and Technology, the State Ethnic Affairs Commission, and the State Sports General Administration of the People's Republic of China using a multistage stratified cluster sampling design. This survey began in 1985 and has been conducted eight times since then, but the test methods used have varied from year to year, e.g., flexibility was tested differently before and after 2000, while the measurement of body mass index was added after 2000. Beginning with the first survey in 1985, children and adolescents in all regions of China have been classified into three levels (upper, middle, and lower) on the basis of their social status, with samples stratified according to their socio-economic status (body height, middle, and low), and later also according to their place of residence (urban and rural). With at least 50 Han Chinese students in each age group included in the survey. The eight surveys selected 14,683 participants in 1985, 2,879 participants in 1991, 7,198 participants in 1995, 2,399 participants in 2000, 10,250 participants in 2005, 7,103 participants in 2014, and 6,333 participants in 2019, with 1 year old as an age group. There were 24 age groups for boys and girls. The survey subjects were all healthy, without disease, disabled, and able to carry out normal physical activities. The studies involving human participants were reviewed and approved by the Medical Research Ethics Committee of the Peking University Health Science Center (IRB00001052-19095). All participants and guardians participated voluntarily and written informed consent by the participant' legal guardian/next of kin were obtained before the survey.

### 2.2 Instruments and procedures

The test work was carried out by trained doctors or physical education teachers. Before the test, the test equipment is calibrated and inspected. The apparatus recommended by Cameron (27) was used by doctors to measure height and weight. The body height is measured with a metal column height-measuring instrument, and the body mass is measured with an electronic weight scale or a lever scale. When measuring body height, the subjects were required to be barefoot, and they were required to wear short sleeves and shorts when measuring their body mass. The measurement results are all accurate to 0.1. Body mass index (BMI) was calculated as body mass in kilograms divided by body height in meters squared [body mass (kg)/body height (m)^2^]. Physical fitness is made up of five motor tests, which are: explosive power (Measured using the standing long jump), speed (measured by 50-m dash), flexibility (measured by sit/stand-and-reach), muscular strength, and cardiorespiratory fitness. Given the disparities in physical fitness according to age and sex, muscular strength was assessed by oblique body pull-ups for boys aged 7–12 years, pull-ups for boys aged 13–18 years, and 1-min sit-ups for girls aged 7–18 years. Cardiorespiratory fitness (CRF) was assessed by 50-m × 8 shuttle run for boys and girls aged 7–12 years, 1000-m running for boys aged 13–18 years, and 800-m running for girls aged 13–18 years. In 1985, flexibility was tested using stand-and-reach. This was later changed to sit-and-reach in 2000 for safety and accuracy reasons.

### 2.3 Statistical analysis

The test results of physical fitness are expressed as the mean ± standard deviation. Sample-weighted linear regression was used to evaluate the secular trend of physical fitness and BMI, with the year as the independent variable and the test result as the dependent variable. The fitting was expressed using *R*^2^ with annual change as *B*. The mean differences among all subgroups were analyzed by one-way analysis of variance (ANOVA) and Bonferroni *post-hoc* test to verify the significance between the survey years every two survey years. The level of statistical significance was set at 0.05. All physical fitness indicators from 1985 to 2019 were divided into four stages: 1985 to 1995 was the first stage, 1995 to 2005 was the second stage, 2005 to 2014 was the third stage, and 2014 to 2019 was the fourth stage. To calculate the change per decade, the formula is as follows: The change per decade (/10a) = (the mean of the next survey year – the mean of the previous survey year)/(the next survey year – the previous survey year) × 10. At the same time, to better understand the differences in physical fitness by sex and age, each fitness is divided into three age categories according to the Chinese educational system: 7–12 years old (primary school), 13–15 years old (junior middle school), and 16–18 years old (junior high school). The data were statistically processed using IBM SPSS 27.0 software and Graph-pad Prism 9.3.1 software.

### 2.4 Ethics statement

The human participant studies underwent review and approval by the Medical Research Ethics Committee at the Peking University Health Science Center (IRB00001052-19095). Participation in the research was voluntary for all individuals and their legal guardians or next of kin provided written informed consent prior to the survey. The research procedures adhered to applicable guidelines and regulations, specifically the detailed protocols outlined in the Chinese National Surveillance on Students' Constitution and Health.

## 3 Results

### 3.1 BMI

**Figure 1:**
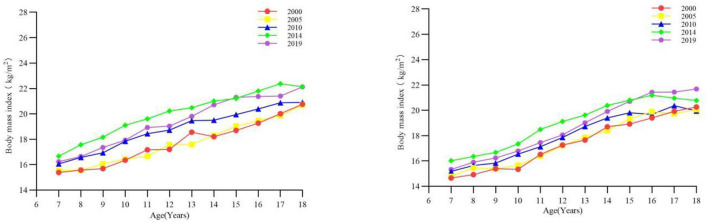
The long-term trend of body mass index of children and adolescents aged 7–18 in Xinjiang from 1985 to 2019 (boys on the **left**, girls on the **right**).

From 2000 onwards, BMI has continued to increase for both sexes in all age groups until 2019 (Figure 1). Except that the BMI of boys aged 7–12 years and 16–18 years decreased from 2014 to 2019 (all *p* < 0.05), the BMI of other age categories in different stages increased (all *p* < 0.05) (Table 1). The BMI of girls aged 7–12 years and 13–15 years from 2014 to 2019 declined, and the BMI in other age categories in the remaining stages increased (all *p* < 0.05) (Table 2).

**Table 1:** Comparison of physical fitness scores of boys in different age groups in Xinjiang from 1985 to 2019.

**Age categories (year)**	**1985(a)**			**1995(b)**			**2005(c)**			**2014(d)**			**2019(e)**			***F***	**Significant post hoe comparisons**	***R* ^2^**	***B***
	**N**	**M**	**SD**	**N**	**M**	**SD**	**N**	**M**	**SD**	**N**	**M**	**SD**	**N**	**M**	**SD**				
**7–12**																			
Body mass index (kg/m^2^)							2,468	16.32	6.8	1,769	18.57	7.81	1,693	17.74	7.2	59.516^***^	c < d, d > e, c < e	0.837	0.148^*^
50-m dash (s)	3,672	10.1	4.7	1,800	9.7	4.6	2,527	10.2	4.7	1,764	10.4	5.0	1,503	10.3	4.7	5.084^***^	a = b, b > c, c = d, d > e, a = e	0.069	0.005
Standing long jump (cm)	3,672	147.6	55.5	1,799	153.5	57.3	1,306	195.4	22.2	1,766	139.3	53.7	1,502	135.3	49.4	15.11^***^	a < b, b > c, c > d, d < e, a = e	0.351	−0.183
Stand/sit-and reach (cm)	3,672	6.3	5.3	1,800	6.3	5.3	2,522	5.8	6.6	1,769	3.6	5.9	1,691	3.8	6.4	112.300^***^	a = b, b > c, c > d, d = e, a > e	0.515	−0.073^*^
Oblique body pull-ups (n)	3,672	14.7	17.4	1,800	29.6	18.4	2,524	22.3	19.0	1,745	22.1	18.5	1,499	22.5	36.9	133.008^***^	a < b, b > c, c = d, d > e, a > e	0.003	0.017
50-m × 8 shuttle run (s)	3,672	112.8	52.2	1,800	114.4	52.4	2,525	125.5	55.9	1,752	130.9	62.1	1,498	132.0	59.2	49.017^***^	a < b, b > c, c < d, d = e, a < e	0.914	0.554^**^
**13–15**																			
Body mass index (kg/m^2^)							1,312	18.3	2.6	900	20.9	3.6	895	20.6	4.2	231.979^***^	c > d, d > e, c > e	0.900	0.190^*^
50-m dash (s)	1,836	8.7	0.7	900	8.1	0.7	1,303	8.4	0.8	897	8.4	1.1	818	8.6	1.1	97.391^***^	a > b, b < c, c = d, d < e, a < e	0.234	−0.009
Standing long jump (cm)	1,836	176.8	33.5	900	202.6	22.7	1,306	195.4	22.2	897	196.2	28.0	820	187.9	27.5	59.293^***^	a < b, b > c, c = d, d < e, a = e	0.319	−0.168
Stand/sit-and reach (cm)	1,836	9.0	5.6	900	9.5	5.9	1,306	7.4	7.1	894	5.4	7.1	895	5.5	6.5	67.987^***^	a = b, b < c, c > d, d < e, a > e	0.531	−0.084^*^
Pull-ups (n)	1,836	3.2	3.0	302	7.0	4.3	1,306	3.2	2.1	897	2.2	2.8	767	8.6	15.4	118.172^***^	a < b, b > c, c = d, d = e, a < e	0.281	−0.005
1,000-m running (s)	1,836	261.2	24.8	899	258.8	25.3	1,292	289.9	37.8	899	300.7	46.5	760	245.8	89.1	331.179^***^	a > b, b < c, c < d, d > e, a < e	0.763	1.233^**^
**16–18**																			
Body mass index (kg/m^2^)							1,280	20.0	2.6	900	22.1	3.8	857	21.6	4.0	132.066^***^	c < d, c < e, d > e	0.835	0.141^*^
50-m dash (s)	1,832	7.9	0.6	900	7.5	0.5	1,304	7.8	0.6	895	7.8	0.9	841	7.7	0.8	88.043^***^	a < b, b < c, c = d, d > e, a < e	0.050	−0.003
Standing long jump (cm)	1,832	181.8	46.4	900	225.7	17.3	1,302	221.8	20.7	896	222.7	24.7	843	220.2	23.3	22.855^***^	a < b, b > c, c = d, d = e, a > e	0.331	0.160
Stand/sit-and reach (cm)	1,832	14.1	5.8	900	13.7	5.8	1,304	11.0	7.3	896	9.5	7.8	855	9.6	6.4	118.622^***^	a = b, b > c, c > d, d = e, a > e	0.785	−0.142^**^
Pull-ups (n)	1,832	7.6	4.6	900	7.9	3.9	1,304	3.8	4.3	895	3.9	3.4	843	2.8	3.6	346.532^***^	a = b, b > c, c = d, d > e, a > e	0.631	−0.136^*^
1,000-m running (s)	1,832	241.1	23.5	900	242.2	24.2	1,298	263.6	33.6	900	272.2	37.1	843	275.0	37.3	303.347^***^	a = b, b < c, c < d, d = e, a < e	0.850	1.046^**^
**All**																			
Body mass index (kg/m^2^)							5,060	17.8	2.9	3,569	20.1	3.9	3,445	19.5	4.1	545.318^***^	d > c, d > e, e > a	0.864	0.157^*^
50-m dash (s)	7,340	9.2	1.3	3,600	8.8	1.3	5,134	9.1	1.5	3,556	9.2	1.6	3,162	9.2	1.6	88.043^***^	a > b, b < c, c > d, d > e, a > e	0.011	−0.002
Standing long jump (cm)	7,340	163.5	36.1	3,599	183.8	38.0	5,136	177.8	39.5	3,559	174.6	44.8	3,165	171.6	44.0	222.575^***^	a < b, b > c, c > d, d < e, a < e	0.396	0.384
Stand/sit-and reach (cm)	7,340	8.9	6.0	3,600	9.0	6.0	5,132	7.5	6.8	3,559	5.5	6.9	3,441	5.7	6.6	250.134^***^	a = b, b > c, c > d, d < e, a > e	0.658	−0.093^*^
Pull-ups (n)	7,340	10.0	12.6	3,600	17.9	16.0	5,134	12.5	15.7	3,537	12.4	15.2	3,109	13.7	27.4	130.909^***^	a < b, b > c, c = d, d = e, a < e	0.01	0.022
1,000-m running (s)	7,340	182.0	72.0	3,599	182.4	71.0	5,115	202.1	81.0	3,551	209.7	85.0	3,101	198.8	82.2	147.562^***^	a = b, b < c, c > d, d = e, a < e	0.891	0.94^**^

N is the sample size; M is the mean; SD is the standard deviation; Conferrable post choc analysis of variance (ANOVA) and sample weighted linear regression are used. ^*^ Indicates *p* < 0.05; ^**^ indicates *p* < 0.01; ^***^ indicates *p* < 0.001.

**Table 2:** Comparison of physical fitness scores of girls in different age groups in Xinjiang from 1985 to 2019.

**Age categories (year)**	**1985(a)**			**1995(b)**			**2005(c)**			**2014(d)**			**2019(e)**			***F***	**Significance post hoe comparisons**	***R* ^2^**	***B***
	**N**	**M**	**SD**	**N**	**M**	**SD**	**N**	**M**	**SD**	**N**	**M**	**SD**	**N**	**M**	**SD**				
**7–12**																			
Body mass index (kg/m^2^)							2,460	15.8	6.4	1,767	17.4	7.3	1,699	16.6	6.6	785.463^***^	d > c, d > e, e > c	0.496	−0.328
50-m dash (s)	3,671	10.6	5.0	1,800	10.0	4.7	2,538	10.7	5.0	1,765	10.8	5.1	1,527	10.7	4.9	7.914^***^	a > b, b = c, c = d, d = e, a = e	0.306	−0.243
Standing long jump (cm)	3,671	139.4	53.0	1,800	144.6	53.6	2,538	134.8	49.6	1,764	129.3	49.7	1,526	128.9	46.4	22.758^***^	a < b, b > c, c < d, d < e, a > e a < b, b > c, c < d, d < e, a > e	0.190	−0.243
Stand/sit-and reach (cm)	3,671	8.3	5.7	1,800	8.6	5.9	2,538	8.8	7.2	1,756	7.4	6.5	1,699	9.0	7.2	15.157^***^	a = b, b = c, d > c, d < e, a < e	0.005	−0.005
1-min sit-ups (n)	3,671	19.8	12.2	1,800	26.2	13.8	2,538	18.4	11.9	1,766	19.8	12.0	1,526	18.6	12.2	158.942^***^	a < b, b > c, c > d, d = e, a = e	0.003	0.092
50-m × 8 shuttle run (s)	3,671	120.3	55.4	1,800	120.2	54.7	2,537	130.7	58.1	1,729	135.6	64.0	1,752	130.9	62.1	46.833^***^	a = b, b < c, c = d, d > e, a > e	0.091	0.185
**13–15**																			
Body mass index (kg/m^2^)							1,274	18.5	2.3	900	20.3	3.2	885	19.8	3.4	125.746^***^	d > c, d > e, e > c	0.763	0.103
50-m dash (s)	1,836	9.8	0.9	898	8.7	0.8	1,296	9.8	0.9	886	9.7	1.1	799	9.6	0.9	276.191^***^	a > b, b < c, c > d, d > e, a > e	0.007	0.003
Standing long jump (cm)	1,836	161.9	18.36	898	167.9	16.1	1,297	161.0	16.1	883	159.0	19.8	800	159.0	18.3	56.320^***^	a < b, b > c, c > d, d > e, a > e	0.201	−0.097
Stand/sit-and reach (cm)	1,860	9.9	5.3	898	11.2	5.3	1,297	9.6	6.7	892	8.2	7.0	884	10.2	7.0	17.611^***^	a = b, b < c, d > c, d < e, a < e	0.109	−0.035
1-min sit-ups (n)	1,836	22.4	9.9	898	30.1	8.6	1,297	24.4	8.6	884	27.6	8.3	800	25.0	9.2	128.018^***^	a < b, b > c, c > d, d = e, a < e	0.000	−0.019
800-m run (s)	1,836	241.9	25.5	898	239.3	22.6	1,289	270.3	32.9	897	272.6	36.8	899	300.7	46.5	292.122^***^	a = b, b < c, c = d, d > e, a = e	0.249	0.679
**16–18**																			
Body mass index (kg/m^2^)							1,270	19.9	2.1	900	21.0	2.8	859	21.5	3.5	47.524^***^	d > c, d > e, e > c	0.037	0.027
50-m dash (s)	1,836	9.8	0.9	900	8.4	1.0	1,282	9.8	0.8	896	9.5	1.0	845	9.5	1.0	378.125^***^	a > b, b > c, c > d, d = e, a > e	0.018	0.005
Standing long jump (cm)	1,836	160.5	18.7	900	171.4	16.2	1,282	165.5	17.3	897	166.1	17.6	845	164.4	19.0	60.878^***^	a < b, b > c, c = d, d > e, a = e	0.074	0.095
Stand/sit-and reach (cm)	1,836	11.9	5.5	900	13.4	5.3	1,281	11.6	6.7	897	11.4	6.5	858	12.8	6.7	17.748^***^	a < b, b > c, d < c, d < e, a < e	0.086	0.037
1-min sit-ups (n)	1,836	20.2	10.3	900	30.3	8.5	1,282	28.1	9.8	895	33.1	8.4	844	29.4	9.5	379.600^***^	a < b, b > c, c > d, d > e, a < e	0.001	0.086
800-m run (s)	1,836	244.0	24.6	900	243.0	25.7	1,277	263.0	4.1	900	263.9	37.6	900	272.2	37.1	120.409^***^	a = b, b < c, c = d, d > e, a < e	0.366	0.649
**All**																			
Body mass index (kg/m2)							5,004	17.6	2.8	3,567	19.0	3.4	3,443	18.7	3.8	218.681^***^	d > c, d > e, e > d	0.382	−0.329
50-m dash (s)	7,343	10.2	1.1	3598	9.3	1.3	5,116	10.3	1.1	3,547	10.2	1.3	3,171	10.1	1.3	453.706^***^	a > b, b < c, c < d, d > e, a > e	0.025	0.004
Standing long jump (cm)	7,343	150.3	22.6	3598	157.1	22.9	5,117	149.1	23.7	3,544	146.0	26.9	3,171	146.0	26.6	125.244^***^	a < b, b > c, c > d, d < e, a = e	0.187	−0.11
Stand/sit-and reach (cm)	7,367	9.6	5.2	3599	10.4	18.4	5,116	9.7	6.3	3,545	8.6	6.4	3,441	10.2	6.6	21.588^***^	a < b, b > c, c > d, d < e, a < e	0.001	−0.065
1-min sit-ups (n)	7,343	20.6	10.3	3598	28.2	9.8	5,117	22.4	10.5	3,545	25.1	10.6	3,170	23.1	10.8	360.643^***^	a < b, b > c, c < d, d < e, a < e	0.001	0.087

N is the sample size; M is the mean; SD is the standard deviation; Conferrable *post choc* analysis of variance (ANOVA) and sample weighted linear regression are used. ^*^ Indicates *p* < 0.05; ^**^ indicates *p* < 0.01; ^***^ indicates *p* < 0.001.

### 3.2 Changes in motor tests

#### 3.2.1 Speed

**Figure 2:**
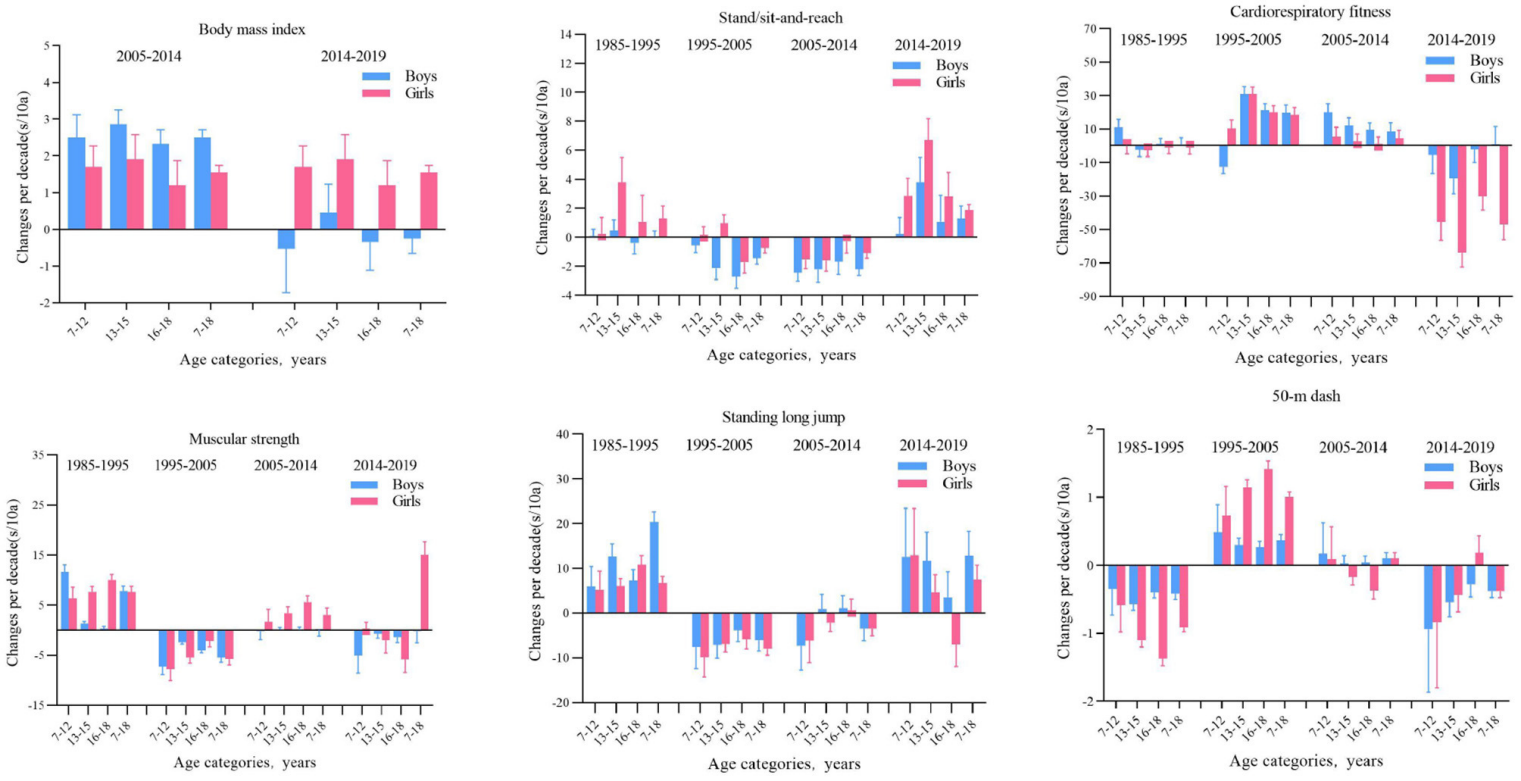
The long-term trend of speed and quality of children and adolescents aged 7–18 in Xinjiang from 1985 to 2019 (boys on the **left**, girls on the **right**).

**Figure 3:**
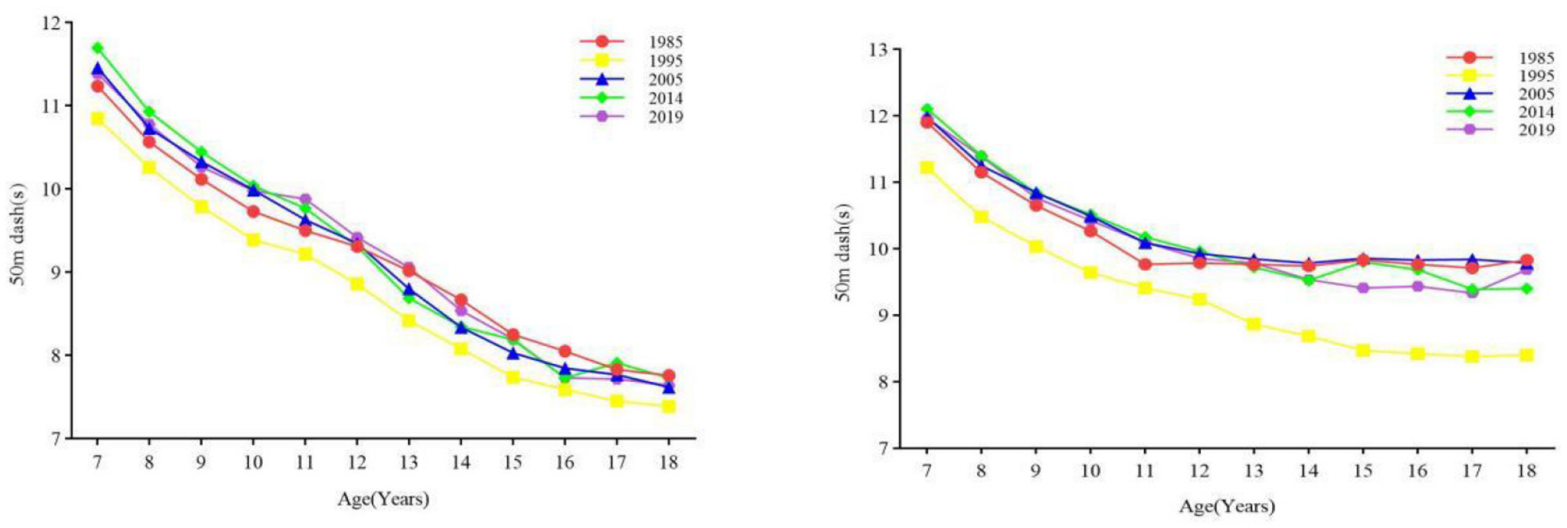
Mean differences (with 95% confidence intervals) in physical fitness test results among boys and girls aged 7–18 years over the years in Xinjiang from 1985 to 2019.

The overall speed of Xinjiang children and adolescents aged 7–18 years worsened but improved in some age groups (Figure 2). The results of one-way ANOVA showed that there was a significant difference in the 50-m dash performance of boys and girls in all age categories between years (all *p* < 0.001) (Tables 1, 2). The speed of boys aged 13–15 and 16–18 years significantly increased from 1985 to 1995 (all *p* < 0.05) and significantly decreased in the other phases and age groups (all *p* < 0.05) (Table 1). The speed of girls aged 7–12, 13–15, and 16–18 years from 1985 to 1995 and girls aged 13–15 and 16–18 years from 2005 to 2014 significantly improved (all *p* < 0.05) and significantly declined (all *p* < 0.05) in the other age groups and periods (Table 2). In terms of the rate of change, in the first stage, the rate of change in the speed of boys and girls aged 7–18 showed an overall upward trend, and the rate of increase in boys was greater than that in girls. In the fourth stage, the speed change of boys decreased, but that of girls increased, and the rate of increase was small (Figure 3).

#### 3.2.2 Explosive force

**Figure 4:**
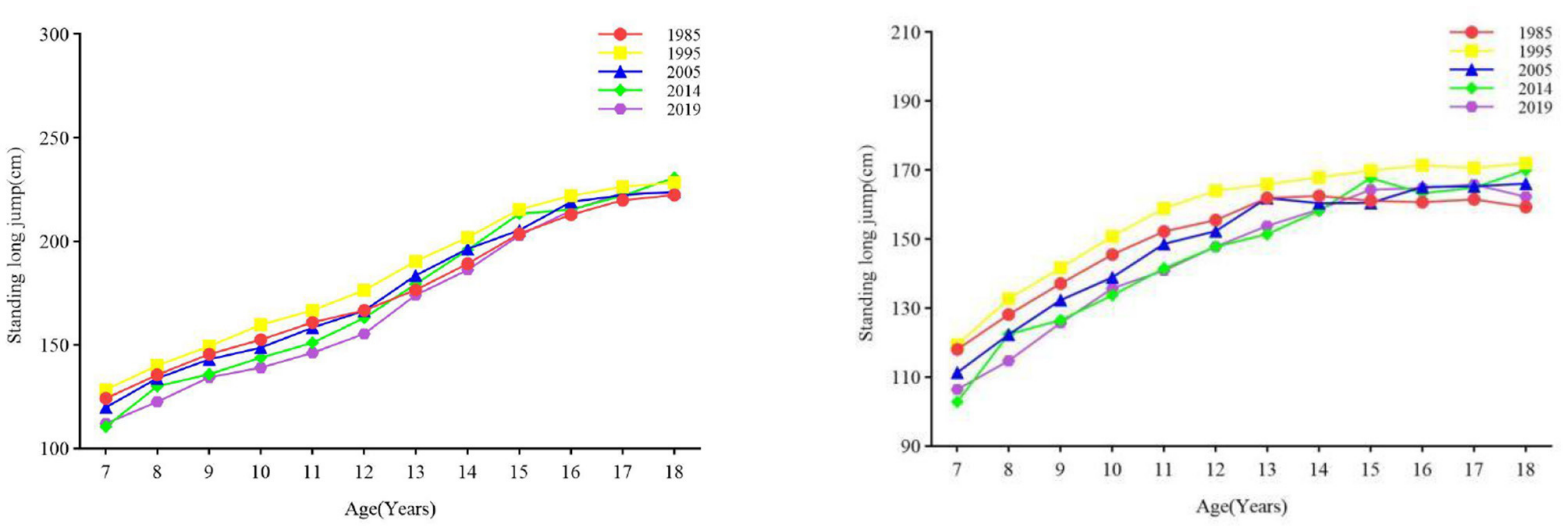
The secular trend of the power of children and adolescents aged 7–18 in Xinjiang from 1985 to 2019 (boys on the **left**, girls on the **right**).

From 1985 to 2019, the overall power of boys and girls decreased (Figure 4). The results of one-way ANOVA showed that there was a significant difference in the standing long jump performance of Boys and girls in the 7–18 age group categories between years (all *p* < 0.001) (Tables 1, 2). From 1985 to 1995, the power of boys and girls aged 7–12, 13–15, and 16–18 increased (all *p* < 0.05), and power decreased in other age groups and phases (all *p* < 0.05). In terms of rates of change, the change in the explosive power quality of boys and girls was the most obvious in the first stage, and the change range of boys was greater than that of girls. From the first stage to the fourth stage, only a small number of boys and girls in the age group had a larger change in explosive power, while the others all declined (Figure 3).

#### 3.2.3 Flexibility

**Figure 5:**
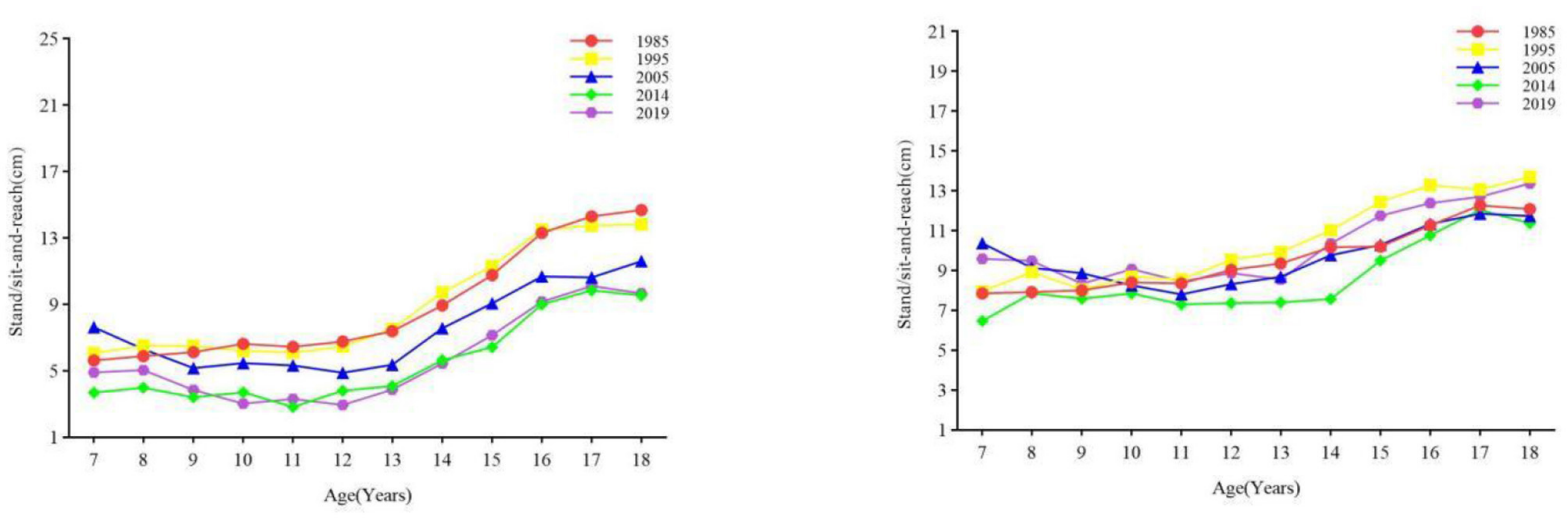
The secular trend of the flexibility of children and adolescents aged 7–18 in Xinjiang from 1985 to 2019 (boys on the **left**, girls on the **right**).

From 1985 to 2019, the flexibility of boys showed an overall downward trend, while that of girls first declined and then increased until 2019, when the flexibility level was almost the same as that in 1985 (Figure 5). The results of one-way ANOVA showed that there was a significant difference between boys and girls in standing/sitting and reaching in the age group of 7–18 years old (all *p* < 0.001) (Tables 1, 2). From 1985 to 2019, the flexibility of boys in all age groups decreased at all stages, among which the differences between 1995–2005 and 2005–2014 were statistically significant (all *p* values < 0.05); from 2014 to 2019, the flexibility of girls aged 7–12, 13–15, and 16–18 all increased (all *p* < 0.05); from 2005 to 2014, the flexibility of girls aged 7–12, 13–15, and 1995. In 2005, the flexibility of girls aged 16–18 all decreased (all *p* values < 0.05). From the perspective of the rate of change, in the first stage, except for girls aged 13–15 and girls aged 16–18 Except for boys, the change range of flexibility of boys and girls in other age groups increased; in the second stage, except for girls aged 7–12 and 13–15 years old, the change range of flexibility of boys and girls in other age groups decreased, and in the third stage, all age groups The range of change in flexibility of both boys and girls decreased, and in the fourth stage, boys and girls in all age groups began to increase again, among which the range of increase was most obvious for girls (Figure 3).

#### 3.2.4 Muscular strength

**Figure 6:**
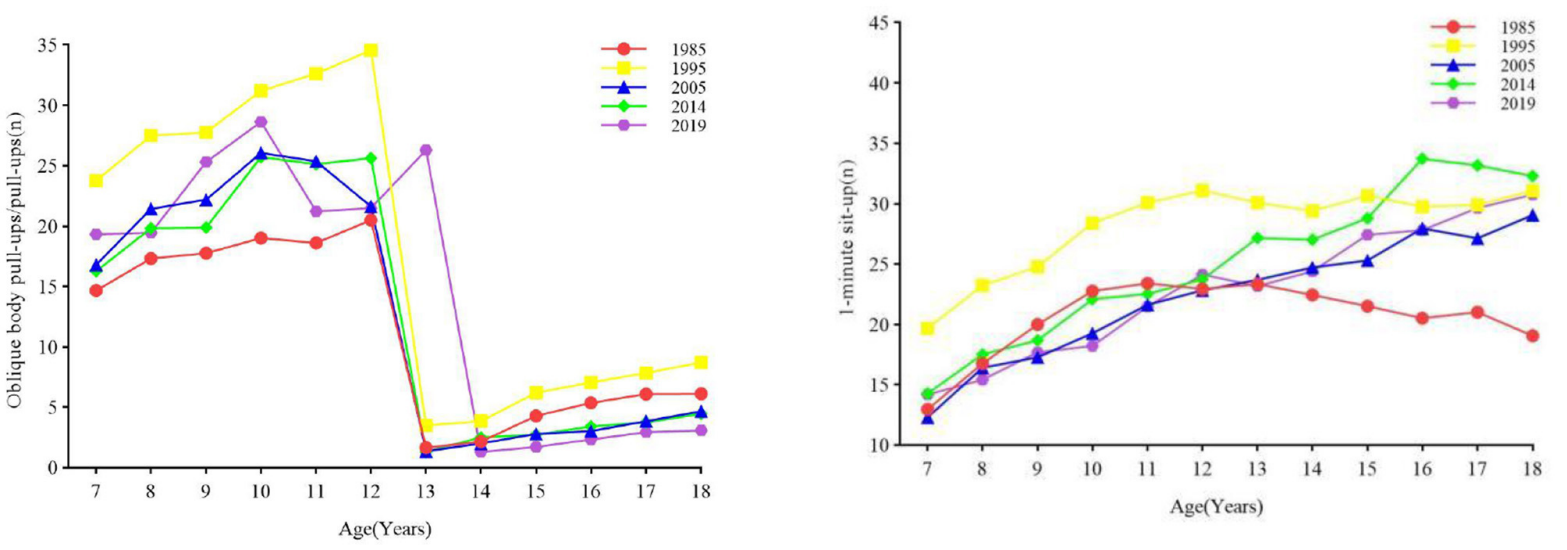
The secular trend of muscular strength of children and adolescents aged 7–18 in Xinjiang from 1985 to 2019 (boys on the **left**, girls on the **right**).

From 1985 to 2019, the overall muscular strength of boys and girls decreased, and the pace of decline of boys was greater than that of girls (Figure 6). From the results of one-way ANOVA, it is evident that there is a significant difference in muscle strength between boys and girls in the age group of 7–18 years (all *p* < 0.001) (Tables 1, 2). From 1985 to 1995 and from 2014 to 2019, the muscular strength of boys aged 7–12 and 13–15 increased (all *p* < 0.05) and decreased in all other age groups (all *p* < 0.05). The muscular strength for girls aged 7–12 years, 13–15 years, and 16–18 years from 1985 to 1995 and from 2005 to 2014 increased (all *p* < 0.05). Obvious than that of boys, while in the fourth stage, girls all decreased, and boys all increased except for the age group of 16–18 (Figure 3). Muscle strength decreased in girls aged 13–15 and 16–18 from 1995–2005 and 2005–2014, and in girls aged 7–12 from 2014–2019 (all *p* < 0.05). The rate of change: In the first stage, the range of change in the muscle strength of boys and girls increased, in the second stage, both decreased, and in the third stage, girls increased The magnitude was more was more obvious than that of boys, while in the fourth stage, girls all decreased, and boys all increased except for the age group of 16–18 (Figure 3).

#### 3.2.5 Cardiorespiratory fitness

**Figure 7:**
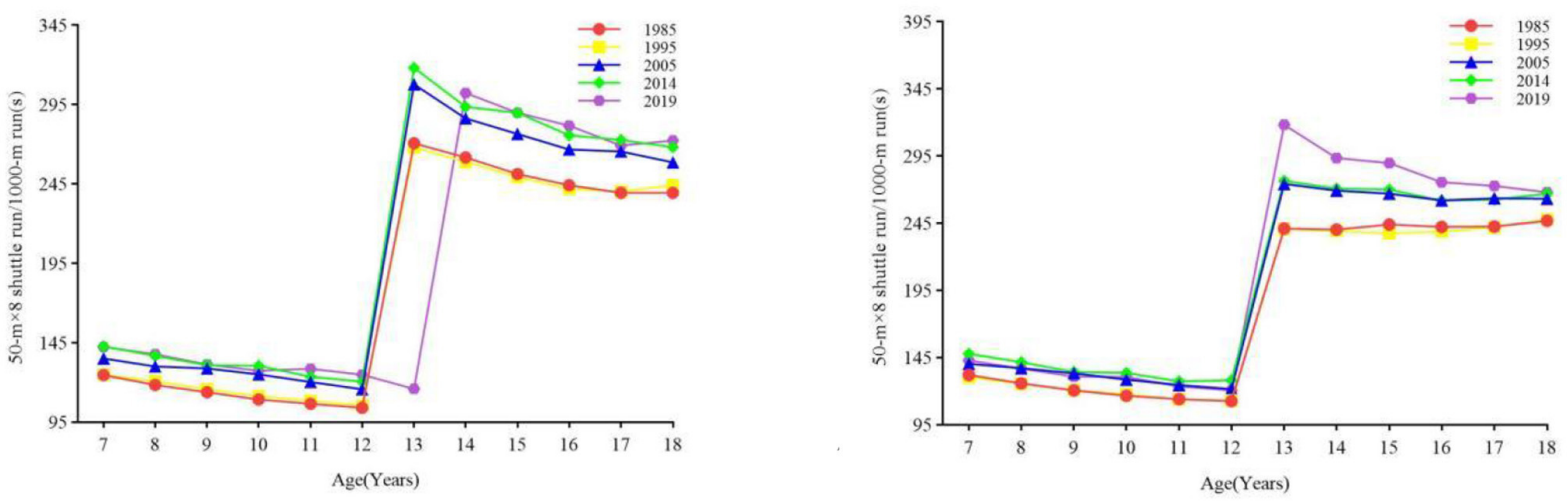
The secular trend of cardiorespiratory fitness of children and adolescents aged 7–18 in Xinjiang from 1985 to 2019 (boys on the **left**, girls on the **right**).

From 1985 to 2019, the overall CRF of boys and girls continued to decrease (Figure 7). The results of one-way ANOVA showed that there was a significant difference in the endurance running performance of boys and girls in all age categories between years (all *p* < 0.001) (Tables 1, 2). From 1995 to 2005 and from 2005 to 2014, the CRf of boys aged 7–12, 13–15, and 16–18 decreased (all *p* < 0.05), while the CRF of boys aged 13–15 in 2014–2019 improved (all *p* < 0.05). The CRF of girls aged 7–12 years, 13–15 years, and 16–18 years from 1985 to 1995 and girls aged 13–15 years and 16–18 years from 2014 to 2019 decreased (all *p* < 0.05). Rate of change: In the first stage, the changing trend of the CRF of boys and girls is relatively stable, without an obvious increase or decrease. The changing trend of the CRF of boys in the 13–15 age group is relatively stable, while the declining trend of the change range of the CRF of girls in the 13–15 age group is more obvious (Figure 3).

## 4 Discussion

The research results show that from 1985 to 2019, the overall physical fitness level of children and adolescents aged 7–18 in Xinjiang worsened. During the period from 1985 to 1995, the speed, power and CRf improved significantly, but from 1995 to 2005, the speed, BMI, power, and CRF declined, and then the downward trend was alleviated to a certain extent in 2005–2014, and some fitness rebounded. Until 2014–2019, only some components of motor abilities in some age groups improved, but most components of motor abilities still worsened.

Consistent with previous research conclusions (17), the results of this study show that the motor abilities of girls aged 7–18 in Xinjiang still needs to be improved urgently from 2014 to 2019, but the difference is that in 2019, the decline in motor abilities of children and adolescents in Xinjiang decreased. The downward trend has been eased, which may be related to the national and local governments' emphasis on the motor abilities of children and adolescents. With the decline in the motor abilities of children and adolescents in China, the Chinese government has actively formulated relevant policies and measures to alleviate the decline in the motor abilities of adolescents. Chinese provinces, cities (autonomous regions and municipalities directly under the central government) actively responded and implemented (28–30). From 2014 to 2019, the overall level of speed quality of male and female students improved, the decline rate gradually decreased, and it began to show an upward trend. This is consistent with the results of relevant research in China (31, 32). These studies have shown that the speed of boys and girls in recent years has improved. There are also studies in other countries that show that the speed of children and adolescents tends to be stable (33–37). The reason for the difference may be different from the level of economic development of each country on the one hand (38). On the other hand, it may also be related to the specific way each country uses to test the program of speed (39, 40), but in general, the determinant of speed is still based on genetics (41, 42). The power of boys and girls aged 7–18 in Xinjiang improved rapidly from 1985 to 1995, and then from 2000 to 2019, the overall power of boys and girls continued to decline, which is consistent with the results of relevant research in China (30, 42–45). The decline in the power of boys and girls is not just a problem in a single region, which is a global problem. The research results of some developed countries also show that (36, 46–48) the power of children and adolescents has worsened. Decreased power is associated with low levels of muscular fitness (i.e., muscular strength, muscular power, and local muscular endurance) and poor exercise capacity in children and adolescents (49–51). Decreased strength in children and adolescents is associated with diminished levels of muscular fitness as well as athleticism. (49–51). We also found that from 1985 to 2019, the flexibility of boys and girls decreased in 1995–2005 and 2005–2014 and improved in 2014–2019. Among them, the flexibility of girls increased the most. This is contrary to the research results in urban areas of China (52), while studies in some developed countries have shown that the flexibility of children and adolescents has continued to decrease in recent years (36, 46–48). Meanwhile, some studies have also shown (53, 54) that there are gender differences in the trend of flexibility of children and adolescents. The flexibility of boys has a secular declining trend, while the flexibility of girls is more stable and has begun to increase in recent years. In terms of muscular strength, compared with 1985, the overall muscular strength of girls in 2019 improved, while that of boys decreased. This is similar to the trend of power of children and adolescents in China (30, 42–45). Studies have shown that the muscular strength of children and adolescents in most European countries has also shown an upward trend, and only a few countries have shown a downward trend, while most developing countries have less research on the secular trend of children and adolescents' muscle strength. There are also differences in testing methods and standards, therefore, different testing methods also have a certain degree of influence on the differences in results. From 1985 to 2019, the BMI of boys and girls continued to increase, which is consistent with the national level in China (30, 42–45). Higher or lower BMI will have a negative impact on the motor abilities of children and adolescents (55–58), and the results of the present study also found that the growth of the BMI of boys was greater than that of girls, especially during puberty (58), which may be the result of adolescent girls paying more attention to their body shape and deliberately maintaining a slim body shape (59, 60). An excessive increase in BMI will lead to a number of cardiovascular diseases, and some studies have shown that the prevalence of obesity is increasing in both sexes in East and South Asia, and the global prevalence of obesity has increased from 0.7% in 1975 to 5.6% in 2016 (61). In contrast, BMI in children and adolescents has plateaued in some high-income regions (61). Physical fitness is a fundamental marker of health in children and adolescents. It can be divided into two components: the health-related component and the skill-related component (62). The health-related component primarily focuses on physical fitness outcomes, while the skill-related component centers on motor skill performance, which is assessed through specific motor skill tests such as cardiorespiratory endurance measured by 800/1,000 m running, muscle strength evaluated through pull-ups and sit-ups, and agility assessed by seated forward bends. Enhancements or declines in sport-specific abilities reflect corresponding changes in physical fitness. Various factors influence physical fitness, with demographic-sociological factors being primary determinants. These factors include age, gender, economic status, education level, dietary habits, etc. (63); since this study did not include indicators related to demographic-sociological factors for analysis, it is not possible to explore these influences in depth; however, it is evident that these demographic-sociological factors significantly impact the physical fitness development of children and adolescents in Xinjiang (16); between 1985 and 2019, there was an overall decreasing trend in the physical fitness of children and adolescents aged 7 to 18 years in Xinjiang, which may be related to the increase in BMI. By 2019, there was an upward trend in the overall BMI levels of children and adolescents in this age group in Xinjiang. The increase in BMI may be attributed to prolonged sedentary behavior, reduced physical activity, and dietary habits (64), and all these factors are likely contributors to the physical fitness levels of children and adolescents aged 7 to 18 years in Xinjiang. However, the declining trend in physical fitness among children and adolescents in Xinjiang was mitigated by 2019, possibly due to the support of national policies. In recent years, with the deterioration of physical fitness among children and adolescents in China, the Chinese government has issued numerous policy documents that have positively influenced the physical fitness and health of this demographic (65).

## 5 Conclusion

This study discussed the secular trend of physical fitness of children and adolescents aged 7–18 in Xinjiang from 1985 to 2019 and found that the overall level of physical fitness of children and adolescents aged 7–18 in Xinjiang worsened. At the same time, there were gender and age differences in the secular trend of physical fitness, while BMI continued to increase. This should attract the attention of relevant local government departments, and policy measures should be actively adopted to alleviate the downward trend of physical fitness and prevent children and adolescents from being overweight or the occurrence of obesity. We should also pay attention to the gender and age differences in the physical fitness of children and adolescents. This study provides a reference for subsequent research on the physical fitness of children and adolescents in Xinjiang and is also a reference for the physical fitness of children and adolescents in different regions of China. However, the influencing factors of the secular trend of physical fitness of children and adolescents aged 7–18 in Xinjiang need to be further explored.

## Data Availability

The original contributions presented in the study are included in the article/supplementary material, further inquiries can be directed to the corresponding author.
